# Deficits in episodic memory retrieval reveal impaired default mode network connectivity in amnestic mild cognitive impairment

**DOI:** 10.1016/j.nicl.2014.02.010

**Published:** 2014-02-27

**Authors:** Cameron J. Dunn, Shantel L Duffy, Ian B Hickie, Jim Lagopoulos, Simon J.G. Lewis, Sharon L. Naismith, James M. Shine

**Affiliations:** a Healthy Brain Ageing Program, Clinical Research Unit, Brain & Mind Research Institute, University of Sydney, NSW, Australia

**Keywords:** Functional magnetic resonance imaging, Resting state functional connectivity, Mild cognitive impairment, Memory, Amnestic, Default mode network

## Abstract

Amnestic mild cognitive impairment (aMCI) is believed to represent a transitional stage between normal healthy ageing and the development of dementia. In particular, aMCI patients have been shown to have higher annual transition rates to Alzheimer's Disease (AD) than individuals without cognitive impairment. Despite intensifying interest investigating the neuroanatomical basis of this transition, there remain a number of questions regarding the pathophysiological process underlying aMCI itself. A number of recent studies in aMCI have shown specific impairments in connectivity within the default mode network (DMN), which is a group of regions strongly related to episodic memory capacities. However to date, no study has investigated the integrity of the DMN between patients with aMCI and those with a non-amnestic pattern of MCI (naMCI), who have cognitive impairment, but intact memory storage systems. In this study, we contrasted the DMN connectivity in 24 aMCI and 33 naMCI patients using seed-based resting state fMRI. The two groups showed no statistical difference in their DMN intra-connectivity. However when connectivity was analysed according to performance on measures of episodic memory retrieval, the two groups were separable, with aMCI patients demonstrating impaired functional connectivity between the hippocampal formation and the posterior cingulate cortex. We provide evidence that this lack of connectivity is driven by impaired communication from the posterior cingulate hub and does not simply represent hippocampal atrophy, suggesting that posterior cingulate degeneration is the driving force behind impaired DMN connectivity in aMCI.

## Highlights

• First trial to explore Default Mode Network (DMN) connectivity between MCI and naMCI
• Amnestic and nonamnestic MCI groups show similar overall DMN intra-connectivity.
• aMCI patients have connectivity deficits related to impaired memory retention.
• Impaired DMN connectivity is driven by deficits in posterior cingulate cortex.
• Alzheimer’s disease pathology likely evolves from PCC to hippocampus

## 1 Introduction

Mild cognitive impairment (MCI) is a clinical state of cognitive decline “greater than expected for an individual's age and education level” (Gauthier et al., 2006) and has been proposed to represent an intermediate stage between normal ageing and dementia (Petersen et al., 2009). The annual rate of progression from MCI to Alzheimer's Disease (AD) is approximately 14%, which is markedly greater than the expected 1–2% annual incidence of AD (Petersen et al., 2009). Thus, MCI has been hypothesised to represent a transitional phenotype for future dementia states (Villain et al., 2008).

Patients with MCI can be further sub-divided based on patterns of cognitive impairment observed on neuropsychological testing. Impaired performance on neuropsychological tests of episodic memory retention and retrieval (Petersen and Morris, 2005) is consistent with an amnestic profile (aMCI) and this sub-group is thought to share a common pathophysiological mechanism with AD (Dubois and Albert, 2004). In contrast, non-amnestic (naMCI) patients have preserved episodic memory and as such are more likely to transition to other forms of dementia (Petersen et al., 2009). In regard to neurobiological markers, patients with aMCI have decreased grey matter volume in the hippocampus (Shi et al., 2009) along with impairments in hippocampus connectivity whilst completing memory tasks (Bai et al., 2009). Furthermore, these differences have been localised to the left hippocampus (Lye et al., 2006), with decreased grey matter volume associated with specific impairments in episodic memory function, as measured by the Rey Auditory Verbal Learning Test (RAVLT) (Hickie et al., 2005). Despite these insights, uncertainty remains concerning the pathophysiological processes underlying aMCI and AD (Sperling et al., 2010).

To delineate the pathophysiological mechanisms underlying aMCI, a number of recent studies have adopted functional neuroimaging approaches (for a review, see Sperling et al., 2010). Earlier studies had focused on task related increases in neural activity as determined using traditional techniques, however more recent approaches have employed resting state functional connectivity MRI (rsfcMRI) measures to examine the coordinated patterns of the brain responses in the absence of explicit tasks. Activity in rsfcMRI is thought to reflect both direct and indirect synaptic activities and provides insight into the information processing capacity of the human brain (Greicius et al., 2009). This method has documented the organisation of the brain into multiple large-scale neural networks (Fox et al., 2005) that retain their functional organisation during task performance (Smith et al., 2009) as well as sleep (Horovitz et al., 2009).

The default mode network (DMN) is one such network and this is of particular interest in aMCI and AD. The DMN has been shown to underpin self-referential and episodic memory capacities (Andrews-Hanna et al., 2010; Spreng and Grady, 2010) and as such, several groups have explored the patterns of intrinsic functional connectivity during the resting state in both MCI and AD (Greicius et al., 2004; Rombouts et al., 2005). Studies have consistently identified reduced intrinsic connectivity amongst MCI patients in the posterior cingulate cortex (PCC), a core hub of the DMN (De Vogelaere et al., 2012). Interestingly, several studies have reported hyperactivation of medial temporal lobe (MTL) structures in MCI patients, yet these findings remain variable (Sperling et al., 2010). Further, increased MTL functional connectivity with the hippocampus is observed during successful memory formation (Ranganath et al., 2005). Impaired DMN activity has been directly related to altered cognitive processing through correlation with neuropsychological data (De Vogelaere et al., 2012). The findings from these studies thus support the notion that the episodic memory impairments in aMCI and AD may be directly related to impairments in connectivity between the MTL and the core regions of the DMN.

To our knowledge, rsfcMRI has not been utilised to compare DMN connectivity between a cohort of patients with aMCI and those with naMCI. We hypothesised that the two groups would differ based on functional connectivity within the DMN, with specific impairments relating to the communication between the MTL and the core regions of the DMN. To explore this hypothesis, we examined the seed-based resting-state functional connectivity in the left hemisphere hubs of the DMN between the aforementioned patient groups. In addition, we sought to determine how these regions coordinated their activity as a function of performance on the RAVLT, a robust test of episodic memory capacity (Andersson et al., 2006).

## 2 Methods

### 2.1 Participants

A total of 57 health seeking older adults meeting criteria for MCI were recruited from the Healthy Brain Ageing Clinic, at the Brain and Mind Research Institute, Sydney, Australia. All participants received a comprehensive medical assessment by an old age psychiatrist, and exclusion criteria included: diagnosed dementia; neurological disease (e.g., Parkinson's, epilepsy); psychosis; prior stroke or head injury (with loss of consciousness >30 min); and, inadequate English for neuropsychological assessment. This study was approved by the University of Sydney Institutional Ethics Committee, and all participants gave written informed consent.

### 2.2 Neuropsychological assessments

As part of a comprehensive assessment battery (Duffy et al., 2014), a neuropsychologist administered the RAVLT (Lezak, 1995), a test which has been linked to hippocampal atrophy (Hickie et al., 2005) and predictive of conversion to dementia in MCI (Andersson et al., 2006). This task required patients to learn a list of 15 unrelated words over 5 trials. The total number of words recalled over the first 5 trials was calculated as a measure of episodic memory encoding (RAVLT_1–5_). After a 20-minute delay, patients were asked to again recall the words, and memory retention was calculated as a percentage of words retained (i.e., trial 7 divided by trial 5 ∗ 100; RAVLT_7/5_). For descriptive purposes, a neuropsychologist also administered the Mini Mental State Examination (MMSE) (Folstein et al., 1983) and the Wechsler Test of Adult Reading as an estimate of predicted IQ (Hartman, 2009). Additionally, patients completed the Geriatric Depression Scale (GDS) (Yesavage et al., 1982).

A diagnosis of MCI was determined using Petersen's criteria requiring cognitive decline of at least 1.5 standard deviations on at least one neuropsychological test, relative to age- and education-adjusted normative data (Petersen and Morris, 2005). Per criteria, each participant was required to have subjective and objective cognitive decline, but with the general preservation of function. MCI diagnoses were consensus rated by an old age psychiatrist and two neuropsychologists, based on clinical profile and neuropsychological assessment and with reference to structural MRI scans where possible. The broad clinical definition of MCI was further categorised into amnestic and non-amnestic subtypes (Petersen and Morris, 2005). In order to be categorised as aMCI, participants were required to demonstrate clear evidence of deficits in memory retention, which was not considered to be merely due to poor encoding. Patients were diagnosed with naMCI if deficits were present on cognitive domains other than memory (e.g., processing speed, working memory, language, visuospatial and executive functioning). As detailed elsewhere (Duffy et al., 2014), the broader neuropsychological test battery included psychomotor speed (Part A of the Trail Making Test [TMT]), working memory (Digit Span subtest of the Wechsler Adult Intelligence Scale — Third Edition), verbal learning and memory (Logical Memory subtest of the Wechsler Memory Scale — Third Edition), language (Boston Naming Test), visuospatial skills (Rey Complex Figure Test, Clock drawing), and executive functioning (Part B of the TMT, DKEFS Stroop and Controlled Oral Word Association Test).

### 2.3 Neuroimaging analysis

#### 2.3.1 Image acquisition

Imaging was conducted on a GE 3 Tesla MRI (General Electric, Milwaukee, USA). T2*-weighted echo planar functional images were acquired in sequential order with repetition time (TR) = 3 s, echo time (TE) = 32 ms, flip angle = 90°, 32 axial slices covering the whole brain, field of view = 220 mm, inter-slice gap = 0.4 mm, and inplane voxel size = 3.4 mm by 3.4 mm by 4 mm thick. A T1-weighted Magnetisation Prepared Rapid Gradient-Echo (MPRAGE) sequence producing 196 sagittal slices with TR = 7.2 ms, TE = 2.8 ms, flip angle = 10°, matrix 256 × 256 and 0.9 mm isotropic voxels was also obtained for localisation of functional resting state loci via co-registration with echo planar functional images. The T1-weighted images were also used to calculate the hippocampal volumes for each participant. A single rsfcMRI run was performed in the scanner by each patient and consisted of patients lying supine with their eyes closed. Patients were also instructed to allow their mind to wander freely.

### 2.4 Resting state functional connectivity fMRI analysis (rsfcMRI)

In order to determine whether the regions identified in the functional analysis formed consistent functional networks, rsfcMRI analyses were applied to task-independent T2*-weighted data. Statistical parametric mapping software (SPM8, Wellcome Trust Centre for Neuroimaging, London, UK) was used for image processing and analysis. Of 127 individual volumes collected from each subject, the first five whole brain scans were discarded to eliminate spurious T2*-equilibration effects. Resting state images were then pre-processed according to a standard pipeline: a) scans were slice-time corrected to the median (17th) slice in each TR; b) scans were then realigned to create a mean realigned image and measures of six degrees of rigid head movements were calculated for later use in the correction of minor head movements; c) due to the increased risk of head movements in this clinical population, each trial was also analysed using ArtRepair (Mazaika, 2007) and trials with a large amount of global drift or scan-to-scan head movements greater than 1 mm were corrected using interpolation. Trials with head movements greater than 3 mm or three degrees of movement were removed from the analysis; d) images were normalised to the T2* image template; e) scans were then smoothed using an 8 mm full-width half-maximum isotropic Gaussian kernel. The mean degree of movement in each of the six motion parameters was subsequently analysed to ensure that there were no significant differences between the two groups.

### 2.5 Seed region definition

**Fig. 1:**
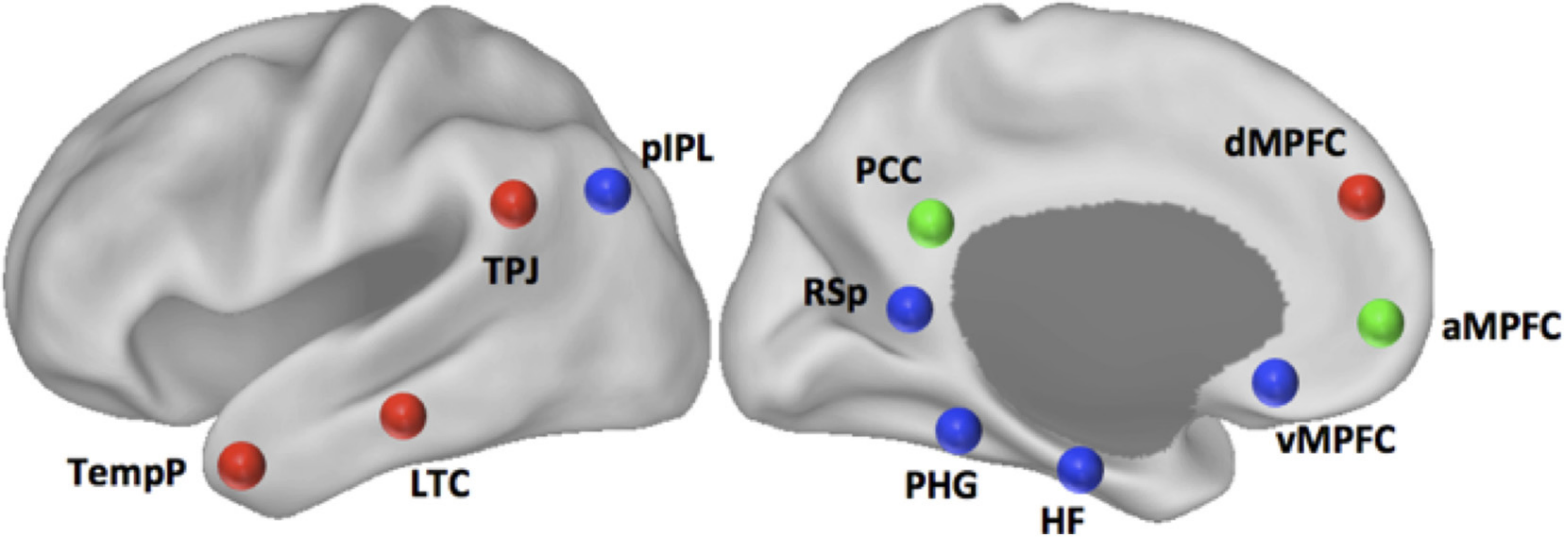
Seed-based regions of interest were drawn around the important hubs within the default mode network (based on the co-ordinates published in a recent study by Andrews-Hanna et al., 2010). The regions are colour-coded according to their inclusion into putative sub-networks: core regions such as the posterior cingulate cortex (PCC) and the anteriomedial prefrontal cortex (aMPFC) are coloured green; dorsomedial regions, including the temporal pole (TempP), the lateral temporal cortex (LTC), the temporoparietal junction (TPJ) and the dorsomedial prefrontal cortex (dMPFC) are coloured red; and the medial temporal lobe subsystem, including the hippocampal formation (HF), the parahippocampal gyrus (PHG), the retrosplenial cortex (RSp) and the ventromedial prefrontal cortex (vMPFC) are coloured blue.

Due to the widespread implication of the DMN in the pathophysiology of aMCI (see Sperling et al., 2010 for a review), a series of regions of interest (ROIs) were defined based on previously published co-ordinates (Andrews-Hanna et al., 2010) (see Fig. 1). Using the Wake Forest University (WFU) PickAtlas toolbox (http://fmri.wfubmc.edu) in SPM8, 8 mm spherical ROIs were created within the three distinct sub-domains of the DMN: the DMN core, containing the posterior cingulate cortex (PCC; −8 −56 26) and the anteriomedial prefrontal cortex (aMPFC −6 53 2); the MTL subsystem, containing the hippocampal formation (HF; −22 −20 −26), the parahippocampal gyrus (PHG; −28 −40 −12), the retrosplenial cortex (RSp; −14 −52 8), the posterior intra-parietal lobule (pIPL: co-ords) and the ventromedial prefrontal cortex (vMPFC; 0 26 −18); and the dorsal medial prefrontal cortex subsystem, containing the dorsomedial prefrontal cortex (dMPFC; 0 52 26), the lateral temporal cortex (LTC; −60 −24 −18), the temporoparietal junction (TPJ; −54 −54 28) and the temporal pole (TempP; −50 14 −40). In order to decrease the number of covariates in the study, ROIs were only drawn from the left hemisphere of each patient according to its association with verbal episodic memory (Lye et al., 2006).

### 2.6 Bivariate correlation

Following the pre-processing steps outlined above, the functional images were imported into the *Functional Connectivity* (‘conn’) toolbox (http://www.nitrc.org/projects/conn) in SPM8 for further data correction. These steps included: a) the application of a temporal band pass filter (0.009–0.08 Hz); and b) the regression of nuisance parameters (and their first temporal derivative), including the six motion parameters extracted from the realignment process; the mean global brain signal; and the signal from 8 mm ROIs placed within white matter and the cerebrospinal fluid. The mean blood oxygenated level dependent (BOLD) signal time course was then extracted from each of the pre-defined ROIs (see below). The time course of each ROI was then correlated with the time course for each of the other ROIs, allowing for the calculation of a correlation coefficient for each ROI using a Pearson's product-moment calculation. These values were then converted into *z*-scores using a Fisher's ‘r-to-z’ transformation and were subsequently used for the statistical comparisons between the two groups.

### 2.7 Bivariate regression

To explore the directionality of any between group differences, we performed a bivariate regression on the ROI pairs that showed a significant degree of functional connectivity difference, either between groups or with respect to neuropsychological performance. In contrast to bivariate correlation, the units in bivariate regression represent ‘effective change’, which is measured as the relative percent signal change in one region with respect to the percent signal change in its target region (Whitfield-Gabrieli and Nieto-Castanon, 2012). This analysis allows for an estimation of the relative strength of information flow in both directions between two ROIs and can inform which ROI is ‘driving’ the relative signal change between two regions (Friston, 2011). Importantly, this measure does not imply causality, but merely that the relative change in BOLD connectivity between two regions is consistently different between two groups (Nieto-Castañón and Fedorenko, 2012; Whitfield-Gabrieli and Nieto-Castanon, 2012). By assessing pairs of ROIs, we are thus able to determine whether one region (a ‘source’) is more likely to predict percent signal change in another region (a ‘target’), but not vice versa. To determine this, we calculated a difference score between pairs of ROIs, resulting in a metric that represented the relative drive from one ROI with respect to its ROI pair. This value was then subjected to a similar analysis pipeline as described below.

### 2.8 Volumetric analysis of the hippocampus

To assess for any significant hippocampal structural differences between the two groups, the individual T1-weighted images for each of the 58 patients were subjected to volumetric analysis using FMRIB's Integrated Registration and Segmentation Tool (FIRST) (Patenaude et al., 2011) implemented in FMRIB's Software Library version 4.0 (FSL; http://fsl.fmrib.ox.ac.uk). The processing pipeline included: skull-stripping using the brain extraction tool (BET) from the FSL; transformation of the T1-weighted data to the Montreal Neurological Institute (MNI) 152 standard space by means of affine transformation based on 12 degrees of freedom; the application of a sub-cortical mask to locate the hippocampus; and segmentation of the hippocampus based on shape models and voxel intensities. An output file, different to that used for the functional analysis, was then generated which contained volumetric data with boundary correction. All hippocampal segmentations were visually inspected to ensure successful registration and segmentation. White matter, grey matter and cerebrospinal fluid volumes were estimated using SIENAX (Smith et al., 2002), which is also part of the FSL. Intracranial volume (ICV) was calculated by adding white matter, grey matter and cerebral spinal fluid volumes for each subject. Individual differences in brain size were corrected for by dividing hippocampal volume by the mean of all subjects' ICV (Raz et al., 2005). To ensure that gross hippocampal volume was not responsible for any ROI-to-ROI connectivity differences, the ICV corrected left hippocampus volume was used as a covariate in the subsequent statistical analyses.

### 2.9 Statistical analysis

Individual *z*-scores representing the relative connection strength between each ROI were extracted from SPM8 and exported to the Statistical Package for Social Sciences (SPSS version 20, IBM Corp.) for further statistical analysis. Initially, we utilised an independent-samples *t*-test to compare for group differences. Following this calculation, we then employed a Pearson's correlation to compare scores on the RAVLT_1–5_ and RAVLT_7/5_ against each of the ROI-to-ROI connection strengths across the entire cohort of MCI patients. We subsequently ran a similar correlation analysis in each sub-group (RAVLT_1–5_ and RAVLT_7/5_vs. ROI-to-ROI connectivity), controlling for hippocampal volume (values were demeaned in each group). Any ROI-to-ROI pairing with significant within-group correlations in each group was then assessed for statistical significance using the Dunn and Clark statistic (ZI) (Dunn and Clark, 1969). To determine the direction of any significant effects, a bivariate regression analysis was employed, in a similar manner to the bivariate correlation analysis. All of the above analyses were two-tailed and used an alpha level of 0.05, with the exception of the Fisher's ‘r-to-z’ test and the Dunn and Clark test, which both used a one-tailed *t*-test. Finally, all significance tests were corrected for multiple comparisons using Bonferroni correction.

## 3 Results

### 3.1 Demographics

As shown in Table 1, there were no significant differences between the aMCI and naMCI patient groups in age, gender, predicted IQ, education level and handedness. As expected, patients with aMCI had significantly lower MMSE scores and recorded poorer performance in the encoding and memory retention components of the RAVLT, compared to those with naMCI.

**Table 1:** Demographic and neuropsychological data between aMCI and naMCI patients (values are mean ± SD).

	aMCI	naMCI	p-Value
Number	24	33	
– Cognitive domain (multiple:single)	22:2	17:16	
Age (years)	74.0 ± 4.9	71.8 ± 4.6	0.087
Education level (years)	14.0 ± 3.7	13.7 ± 3.0	0.730
Predicted IQ	107.2 ± 9.8	108.6 ± 7.5	0.557
Gender (males:females)^a^	7:17	14:19	0.305
Handedness (right:left)^a^	23:1	31:2^b^	0.752
Geriatric Depression Scale (15 point scale)	3.5 ± 3.6	4.4 ± 4.7	0.428
Mini Mental State Exam (MMSE)	27.2 ± 1.9	28.5 ± 1.1	0.006
Rey Auditory Verbal Learning Task (RAVLT)			
– Encoding (RAVLT_1–5_) (%)	31.7 ± 7.6	41.0 ± 8.1	0.001
– Retention (RAVLT_7/5_) (%)	21.1 ± 20.9	72.9 ± 20.1	0.001
Volumetric analysis — ICV corrected (mm^3^)			
– Left hippocampus	2884.2 ± 619.9	3199.9 ± 538.3	0.052

a Chi-squared.

b One naMCI subject was ambidextrous.

### 3.2 Volumetric analysis of the hippocampus

Patients with aMCI had significant volume loss in the left hippocampus (*t*= 2.05; *p*< 0.05) when compared to patients with naMCI.

### 3.3 Resting state connectivity analysis — bivariate correlation

After correction for multiple comparisons, there were no significant differences in any of the ROI-to-ROI connectivity strengths between the two groups. In addition, none of the ROI pairings showed a statistically significant correlation with RAVLT_1–5_ or RAVLT_7/5_ scores across the entire cohort of MCI patients.

**Fig. 2:**
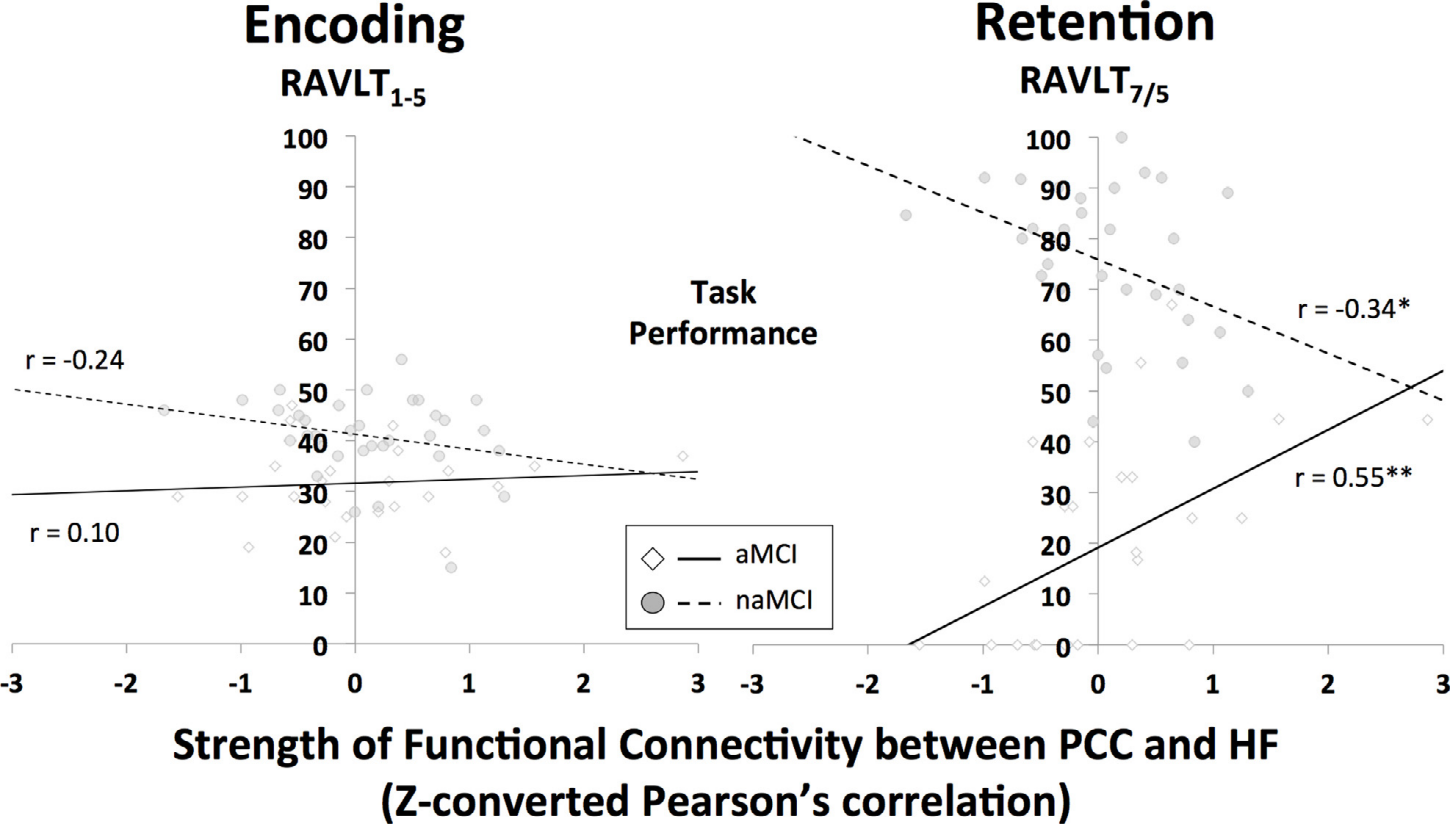
Posterior cingulate cortex:hippocampal formation (PCC:HF) functional connectivity vs. Rey Auditory Verbal Learning Task (RAVLT) in amnestic (aMCI) and non-amnestic (naMCI) mild cognitive impairment patients: left panel — RAVLT_1–5_ encoding score (ZI = 1.2, *p*= 0.2); right panel — RAVLT_7/5_ retention score (ZI = 3.4, *p*< 0.001). Key: * — *p*< 0.05; ** — *p*< 0.005.

When assessing the relationship between RAVLT_7/5_ scores and DMN connectivity in the cohort of patients with aMCI, poor episodic memory retrieval was related to impairments in connectivity strength between the PCC–HF (*r*= 0.552, *p*< 0.005), RSp–HF (*r*= 0.498, *p*< 0.05), TPJ–vMPFC (*r*= 0.448, *p*< 0.05), PCC–LTC (*r*= 0.437, *p*< 0.05) and RSp–PHC (*r*= 0.429, *p*< 0.05). In naMCI patients, significant correlations were found between the dMPFC–pIPL (*r*= 0.502, *r*< 0.005), PCC–LTC (*r*= 0.370, *p*< 0.05), and TPJ–HF (*r*= –0.429, *p*< 0.05). However, only the impaired connectivity between the PCC–HF in aMCI patients survived correction for multiple comparisons. In addition, the connection strength between these two DMN hubs showed opposing directionality between the two groups of MCI patients (aMCI: *r*= 0.552, *p*< 0.005; naMCI: *r*= –0.339; *p*< 0.054) (see Fig. 2). Furthermore, the difference between these two correlations remained strongly significant (ZI = 3.42, *p*< 0.001), even after Bonferroni correction for multiple comparisons. No such relationship was seen for RAVLT_1–5_ scores (see Fig. 2).

### 3.4 Resting state connectivity analysis — bivariate regression

**Fig. 3:**
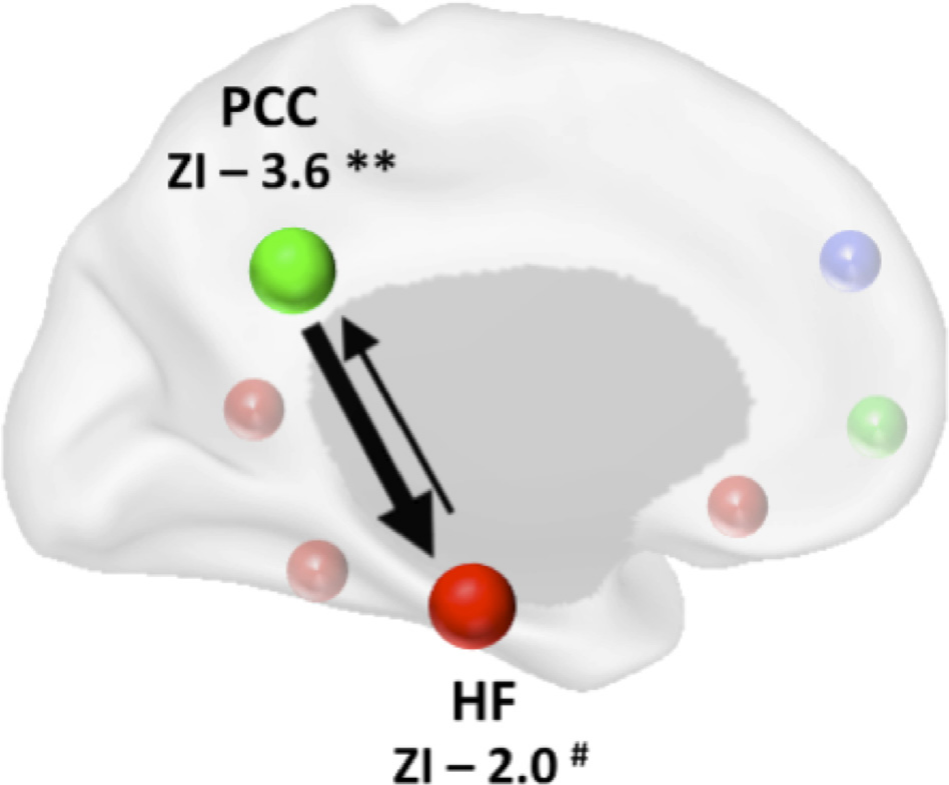
Results from the bivariate regression analysis — the percent signal change in the posterior cingulate was a strong predictor of decreased activity in the hippocampal formation (ZI = 3.6, *p*< 0.001). Although there was a statistical trend for significance in the opposite direction (ZI = 2.0, *p*= 0.089), the difference score between the two groups was significantly larger for the PCC than for the HF (ZI = 1.6, *p*< 0.05).

When exploring the directionality of the impaired connectivity between the HF and the PCC, patients with aMCI showed a significant increase in the percent signal change driven by the PCC (*t*= 2.12, *p*< 0.05). When exploring the nature of the percent signal change with respect to episodic memory consolidation in patients with aMCI, the strength of the PCC connectivity was significantly positively correlated with performance on the RAVLT_7/5_ (*r*= 0.583, *p*< 0.001). Furthermore, patients with naMCI showed an opposite effect (*r*= –0.278, *p*< 0.05) and the difference between the two correlations was statistically significant (ZI = 3.6, *p*< 0.001), suggesting that decreased influence of the PCC over the HF was predictive of episodic memory impairment in aMCI but not naMCI patients (see Fig. 3). Although there was a statistical trend for significance in the opposite direction (ZI = 2.0, *p*= 0.089), the results did not survive correction for multiple comparisons. Finally, the difference score between the two groups was significantly larger for the PCC than for the HF (ZI = 1.6, *p*< 0.05).

## 4 Discussion

To our knowledge, this is the first study to show that MCI subtypes can be discriminated on the basis of specific impairments in DMN resting state functional connectivity and cognitive ability. The differences in default mode connectivity between the two groups were only evident after accounting for impaired performance on tasks probing episodic memory retention, a characteristic that defines patients with aMCI (Greicius et al., 2004; Petersen et al., 2009; Rombouts et al., 2005; Sperling et al., 2010). Our results are strongly aligned with the known impairments in MTL connectivity within the DMN in aMCI patients (Sperling et al., 2010) and extend recent studies that suggest a functional disconnection between the hippocampus and PCC of the DMN during episodic retrieval in aMCI patients (Bai et al., 2009; Huijbers et al., 2011; Ranganath and Ritchey, 2012).

The hippocampus and the PCC are connected anatomically, and functional interactions between the two structures are presumed to underlie normal episodic memory capacities (Ranganath and Ritchey, 2012). The hippocampus itself is a heavily interconnected structure that connects the MTL to a number of cortical and subcortical structures involved in episodic memory formation and retrieval (Jeffery, 2007). The role of the hippocampus in memory is well established, as reflected by lesion studies that report severe amnesia following loss of its structural integrity (Stephan et al., 2012). Moreover functional studies have demonstrated that activity within the hippocampus is also strongly linked to episodic memory retrieval (Ryan et al., 2001) and this relationship is further highlighted in patients with aMCI who have decreased hippocampal grey matter volume, particularly in the dorsal outflow regions, such as the CA1–2 transition zone (Mueller et al., 2010; Pluta et al., 2012). The results from our current study corroborate these previous findings and indeed demonstrate that resting state connectivity in aMCI remains impaired even after accounting for the degree of hippocampal atrophy. This suggests that the impairments are due to a relative disconnection between the two structures, an interpretation that is consistent with previous research (Fellgiebel et al., 2006; Rose et al., 2011; Villain et al., 2008).

The PCC also assumes an important role in episodic memory (Wagner et al., 2005) and has been identified as a key member of the posteriomedial memory system, a neural network encompassing the DMN that has been shown to underlie the processing of ‘contextual’ memory cues, such as the location in which an item was learned (Ranganath and Ritchey, 2012). The posterior medial system interacts dynamically with an anterior medial system, which processes the contents of episodic memory (such as the specific category of a stimulus) and includes cortical territories in the MTL as well as the amygdala (Ranganath and Ritchey, 2012). In this model, the hippocampus is proposed to facilitate information transfer between the two systems, acting as a ‘dynamic integrator’ of episodic memory. It can be conjectured that dysfunctions in the connectivity between the hippocampus and the PCC might manifest as difficulties in incorporating content into a contextual framework, and thus consistent with functional impairments (as reflected by impaired performance) on neuropsychological tasks, such as RAVLT_7/5_. This interpretation is also supported by recent imaging studies that have reported episodic memory retrieval is specifically associated with increased coupling between the DMN and medial temporal cortical regions (Huijbers et al., 2011; Vannini et al., 2011), although the precise neuroanatomical mechanisms underlying these interactions remain unclear (Ward et al., 2014).

The results of this study fit in with our current understanding of the pathophysiological mechanisms underlying the conversion from healthy ageing to AD. Although there is now compelling evidence that supports the accumulation of amyloid and tau pathology in the DMN (particularly in the hippocampus and PCC) (Buckner et al., 2005; de Calignon et al., 2012), debate still remains as to which of these regions is driving the pathophysiological processes (Villain et al., 2008). The results of our bivariate regression analysis provide evidence that the PCC activity is responsible for the subsequent decreased connectivity between the PCC and the hippocampus. In addition, the impairments in functional connectivity were independent of the degree of atrophy seen in the hippocampus, a factor that has previously been shown to differ between aMCI and naMCI patients (Vos et al., 2013). Thus, the results of our analysis strongly support the notion that the pathological degradation in the DMN in aMCI is driven by impairments in the PCC. This result is well aligned with findings from PET (Buckner, 2004), fMRI (Buckner et al., 2005) and MRS studies (Lagopoulos et al., 2013). However, longitudinal studies that explore the evolving nature of amyloid and tau pathology, along with the atrophic and disconnection-related side-effects, will be required before the precise trajectory can be determined.

Functional connectivity measures are thought to reflect the sum of both direct and indirect synaptic connections between neural hubs (Greicius et al., 2009; Sperling et al., 2010). It is not clear from our results whether the decreased functional connectivity between the hippocampus and the PCC manifests through direct or indirect impairments in white matter connectivity. Indeed, these two regions are known to have both direct and indirect synaptic connections, the latter mediated through the fornix and its connections to the mammillary bodies and the anterior thalamic tract as part of the circuit of Papez (Granziera et al., 2011; Hornberger et al., 2012; Kwon et al., 2010). Interestingly, patients with AD have been shown to suffer from changes in white matter integrity in the circuit of Papez (Hornberger et al., 2012), and the degree of this impairment has also been shown to correlate significantly with impaired hippocampal volume as well as decreased glucose metabolism in the PCC (Villain et al., 2008). However, other studies exploring white matter integrity in aMCI have shown specific impairments in the rostral portions of the posterior cingulum bundle (Lim et al., 2012), possibly reflecting a progressive loss of synaptic connections between the PCC and hippocampal formation. Future studies employing diffusion weighted imaging should seek to determine whether between-group differences in episodic memory retention and retrieval within an MCI cohort could be explained by degradation of either direct or indirect white matter connectivity, or both.

The results of this study also raise questions about the pathophysiology underlying naMCI. Although impaired connectivity between the HF and PCC was strongly associated with poor episodic memory retrieval in amnestic MCI patients, a trend towards the reverse relationship was observed in naMCI patients (i.e., worse retrieval predicted increased connectivity between PCC and HF). This may suggest that those non-amnestic individuals with borderline episodic memory retrieval capacity possess the ability to integrate information through the DMN, a trait that may distinguish them from patients with amnestic MCI. Further, individuals with naMCI may suffer from impaired co-ordination between neural regions that comprise one of the other major attentional networks, such as the dorsal attention network (Corbetta and Shulman, 2002; Fox et al., 2006) or the frontoparietal control network (Vincent et al., 2008). These networks are involved in the goal-directed control of exogenous attention as well as the manipulation of the contents of working memory. Indeed, there is evidence from fMRI to support this notion (Machulda et al., 2009), however future experiments exploring the connectivity of each of these networks in both aMCI and naMCI patient groups will be required to help address these questions.

## 5 Conclusion

The results of this study represent the first direct exploration of DMN connectivity impairments between patients with aMCI and those with naMCI. As predicted, patients with aMCI showed specific deficits in the connectivity between the hippocampal formation and the PCC related to their impaired performance on a task designed to test episodic memory retention. Subsequent analysis demonstrated that the impaired connectivity amongst aMCI patients was driven by deficits in the posterior cingulate hub. This finding is consistent with the notion that the pathological process underlying AD begins in the PCC, before extending to impair the hippocampus, and ultimately, the medial prefrontal cortex. Future studies need to investigate the associations between both neuronal and glial metabolites, as well as microstructural white matter tract changes, in the pathophysiological evolution of AD pathology underlying episodic memory dysfunction. Finally, investigating resting state connectivity alongside underlying metabolic disturbances (as determined by PET) might shed further light on the pathophysiological mechanisms that underpin MCI and more importantly the transition between normal ageing and AD.
